# Safety and efficacy of extracorporeal CO_2_ removal combined with continuous renal replacement therapy in patients presenting both acute respiratory distress syndrome and acute kidney injury

**DOI:** 10.1186/cc14355

**Published:** 2015-03-16

**Authors:** J Allardet-Servent, M Castanier, T Signouret, A Lepidi, R Soundaravelou, J Seghboyan

**Affiliations:** 1 Hopital Européen Marseille, France

## Introduction

Pulmonary overdistension has been observed in 33% of patients with acute respiratory distress syndrome (ARDS) despite low tidal volume (6 ml/kg ideal body weight) ventilation [1]. Tidal volume (VT) reduction from 6 to 4 ml/kg attenuates overdistension but is associated with hypercarbia [2]. We thought to combine extracorporeal CO_2_ removal (ECCO_2_R) with continuous renal replacement therapy (CRRT) through the insertion of an oxygenator membrane within the hemofiltration circuit in patients presenting both ARDS and acute kidney injury (AKI).

## Methods

A first set of measurement was performed at 6 ml/kg before and after ECCO_2_R. Twenty minutes later, VT was reduced to 4 ml/kg for the remainder of the study period (72 hours). Ventilator settings were those of the ARMA trial. The CRRT mode was hemofiltration with 33% of predilution. Ultrafiltration was adjusted to achieve a filtration fraction of 15%. Sweep gas flow was constant at 8 l/minute. The primary endpoint was a 20% reduction of PaCO_2_ at 20 minutes after initiation of ECCO_2_R.

## Results

Eight patients were studied. Age was 69 ± 11 years, SAPS II was 68 ± 9 and SOFA score was 13 ± 4 at inclusion. Blood flow, at the inlet of the oxygenator membrane, was 400 ± 4 ml/minute. CO_2_ removal rate was 84 ± 4 ml/minute. Initiating ECCO_2_R, at 6 ml/kg, induced a mean PaCO_2_ reduction of 17% (41 ± 5.5 to 33.9 ± 5.6 mmHg, *P *< 0.001). Then, lowering the VT to 4 ml/kg induced a mean PaCO_2_ increase of 25% (33.9 ± 5.6 to 42.6 ± 8 mmHg) and a mean PaO_2_/FIO_2_ ratio increase of 8% (176 ± 63 to 190 ± 61). Minute ventilation decrease from 7.4 ± 1.6 to 5 ± 1.2 l/minute. Respiratory system compliance did not vary. No major complications were observed.

## Conclusion

Combining ECCO_2_R and CRRT in patients with ARDS and AKI is safe and feasible through the insertion of an oxygenator membrane within a RRT circuit.
